# Towards Automated Testing of Kynurenine for Point-of-Care Metabolomics

**DOI:** 10.3390/mps8030056

**Published:** 2025-06-01

**Authors:** Dipanjan Bhattacharyya, Marcia A. LeVatte, David S. Wishart

**Affiliations:** 1 Department of Biological Sciences, University of Alberta, Edmonton, AB T6G 2E9, Canada; 2 Department of Computing Science, University of Alberta, Edmonton, AB T6G 2E8, Canada; 3 Department of Laboratory Medicine and Pathology, University of Alberta, Edmonton, AB T6G 1C9, Canada; 4 Faculty of Pharmacy and Pharmaceutical Sciences, University of Alberta, Edmonton, AB T6G 2H7, Canada

**Keywords:** kynurenine, chemical assay, diazotization, urine, serum, LC-MS/MS

## Abstract

Our objective was to develop a simple, low-cost colorimetric assay to detect kynurenine (L-Kyn) in human biofluids, that would be compatible with a point-of-care (POC) system being developed in our lab. Elevated L-Kyn is associated with many pathological conditions. However, current detection methods are expensive, time-consuming, and unsuitable for resource-limited settings. Existing colorimetric L-Kyn assays lack specificity, require unusual reagents, or lack sensitivity, hindering their practical application. Here we report a two-step diazotization-based colorimetric assay that produces a red chromophore upon reaction with L-Kyn. To reduce background interference, we used dilution and anion exchange chromatography for urine samples and acid precipitation for serum samples. The assay detected 5–300 μM L-Kyn in urine (lower limit of detection (LLOD) 1.34 μM) and 5–125 μM L-Kyn in serum (LLOD 1.24 μM). Correlation studies achieved strong linearity (R^2^ = 0.98 for spiked urine, 0.99 for spiked serum) and were highly correlated (>0.95) to liquid chromatography tandem mass spectrometry (LC-MS/MS) concentrations. Bland–Altman analysis confirmed agreement between L-Kyn assay and LC-MS/MS methods. To our knowledge, this is the first application of a diazotization reaction for L-Kyn quantification at physiologically relevant levels. The assay is now being ported to a low-cost, automated POC biosensor platform.

## 1. Introduction

The kynurenine pathway (KP), discovered in 1853 [1], is the primary route of tryptophan (Trp) metabolism, accounting for approximately 99% of ingested Trp breakdown [1,2,3]. Oxidation of Trp, catalyzed by indoleamine 2,3-dioxygenase (IDO) and tryptophan 2,3-dioxygenase (TDO), produces L-kynurenine (L-Kyn), the pivotal metabolite of the KP [4,5]. As an essential metabolite for many pathways and processes, L-Kyn plays a key role in immune regulation, brain function, and inflammation. It has also been implicated in a variety of diseases including neurodegenerative and psychiatric diseases [6,7,8], age-associated neuroendocrine disorders [9], immunological disorders [10,11], and cancer [12,13,14,15,16]. In many cases, these disorders are associated with elevated biofluid levels of L-Kyn.

For instance, in serum or plasma, increased levels of L-Kyn as well as elevated L-Kyn/Trp ratios have been observed in patients with Parkinson’s disease [17,18], multiple sclerosis [19], Tourette’s syndrome, and encephalopathies [20] as well as clinically diagnosed anxiety [20]. Likewise, other conditions such as major coronary events and stable coronary artery disease [21,22], irritable bowel syndrome (IBS), ulcerative colitis, Crohn’s disease [23,24], and renal failure [17,25] are also characterized by elevated serum/plasma L-Kyn concentrations or elevated L-Kyn/Trp ratios. High urinary L-Kyn/Trp ratios have also been shown to be diagnostic for urinary tract infections in children [26] while elevated urinary L-Kyn was also observed with major coronary events and stable coronary artery disease [21,22,27], colorectal cancer (CRC) [13], lung cancer [14], and prostate cancer [15]. L-Kyn appears to function as an oncometabolite as cancer cells exhibit increased Trp uptake and metabolism, resulting in elevated L-Kyn levels that activate the transcription factor aryl hydrocarbon receptor (AHR), promoting cancer cell growth [16]. Increased TDO and IDO1 activities lead not only to excessive L-Kyn production but also facilitates invasive tumor growth and tumor cell survival [28,29]. Consequently, detecting elevated levels of L-Kyn in biofluids is crucial for diagnosing, monitoring disease progression, and evaluating therapeutic responses.

Despite its widely known and potential clinical significance, the measurement of L-Kyn in biofluids is not routinely performed. This is because quantifying L-Kyn is challenging due to its relatively low abundance. For instance, normal levels of L-Kyn are <20 µM in urine and <5 µM in serum/plasma, while pathological levels are typically >30 µM in urine and >7 µM in serum/plasma [13,15,30,31,32,33]. As a result, L-Kyn detection and quantification methods must use relatively expensive and highly specialized equipment including high-performance liquid chromatography (HPLC) coupled with ultraviolet (UV), fluorescence, electrochemical, or mass spectrophotometric detection.

Currently, the gold standard for L-Kyn identification and quantification in serum and urine is liquid chromatography mass spectrometry (LC-MS) [13]. While generally less precise and less sensitive, HPLC systems equipped with UV and/or fluorescence detectors can also be used for L-Kyn quantification [34]. Recently, electrochemical detection using carbon nanotube (CNT)-based electrodes has been shown to be effective in detecting and quantifying L-Kyn in plasma samples [35]. Likewise, enzyme-linked immunosorbent assays (ELISAs) have now become available, but these are relatively expensive, multi-step processes (e.g., BA E-2200R, ImmuSmol, CAD 900). The fact that all these methods require specialized equipment, highly trained personnel, and significant financial resources has put L-Kyn testing largely out of reach in most clinical labs and completely out of reach in resource-limited settings found in rural regions or developing countries. Simple, low-cost methods for L-Kyn detection are urgently needed.

One approach to reduce costs and simplify L-Kyn testing would be through the use of chemically based, colorimetric assays. It has been known for some time that aromatic-amine-based reagents such as *p*-dimethylaminobenzaldehyde (DMAB or Ehrlich’s reagent) will react with L-Kyn to produce a colored product. However, the reaction lacks specificity since DMAB also reacts with proteins (found in serum/plasma) as well as other abundant metabolites found in biofluids such as urea [36] and urobilinogen [37]. Likewise, the detection limits for DMAB assays for L-Kyn do not match observed physiological levels. A proof-of-principle colorimetric assay has been developed that uses a coumarin-based fluorescent chemosensor [38]. This method can detect clinically relevant ranges of L-Kyn, but it was only tested in chemically simple, synthetic urine and not on real (chemically complex) urine samples. Likewise, the difficult synthesis of this coumarin derivative, combined with lack of commercial availability, limits its practical application. To date, no simple, accessible, chemical colorimetric assay has been developed that can detect physiologically relevant concentrations (low µM range) of L-Kyn in common biofluids.

Here we report a novel chemical method for detecting and quantifying physiologically relevant levels of L-Kyn in common biofluids using a diazotization reaction. Diazotization is a chemical reaction in which a primary aromatic amine reacts with nitrous acid to form a diazonium salt, which is useful in dye production because diazonium compounds readily undergo coupling reactions with aromatic compounds to form azo dyes, known for their vibrant colors and stability. Such a colorimetric diazotization reaction has not previously been applied to L-Kyn detection. The optimized protocol removes interfering agents from urine, serum, and plasma and rapidly detects 5 μM to 300 μM L-Kyn using inexpensive, readily available reagents. This assay was further optimized to make it compatible with an automated, point-of-care (POC) metabolomic device being developed in our lab that uses colorimetric reactions to detect up to 20 different high-value metabolites in human biofluids [39,40,41,42]. Our ultimate goal is to enable simple, low-cost, and rapid detection of L-Kyn in clinical and field settings.

## 2. Materials and Methods

### 2.1. Materials

L-kynurenine (98%), sodium hydroxide (NaOH pellets, ACS grade, 97%), lithium hydroxide monohydrate (LiOH•H_2_O, 98%), potassium hydroxide (KOH pellets, ACS grade, 85%), hydrochloric acid (HCl, 37%), glacial acetic acid (99%), sodium nitrite (NaNO_2_, 97%), β-naphthol (2-naphthol, 99%), *p*-toluenesulfonic acid monohydrate (*p*-TsOH•H_2_O, 98.5%), potassium acetate (KOAc, 99%), potassium phosphate monobasic, potassium phosphate dibasic, 2,2-dimethyl-2-silapentane-5 sulfonate (DSS-d_6_), D_2_O (99.9%), 9-phenanthrol, 8-hydroxyquinoline, 5-hydroxynaphthalene-1-sulfonic acid, naphthalene-1,5-diol, naphthalene-2,6-diol, 2-aminoisobutyric acid, allantoin, arabinitol, ascorbic acid, citric acid, creatinine, glucose, ethanolamine, glycine, glycolic acid, hippuric acid, cysteine, glutamine, histidine, tryptophan, tyrosine, mannitol, tartaric acid, taurine, urea, lithium acetoacetate, and Amberlite^®^ IRA 400 anion exchange resin (chloride form, 16–50 mesh) were all purchased from Sigma-Aldrich (Oakville, ON, Canada). 2-Chloropyrimidine-5-carboxylic acid (98%) was purchased from ArkPharm (Libertyville, IL, USA). The Bradford assay was purchased from Bio-Rad (Hercules, CA, USA). Absolute ethanol (EtOH) was purchased from Greenfield Global (Brampton, ON, Canada). Three kilodalton (kDa) Amicon filters, PCR® strip tubes, microfuge tubes (1.5 mL and 2 mL), Falcon tubes (15 mL and 50 mL), 96-well plates (BioLite 96 Well Multidish), and pipette tips were purchased from Thermo Fisher Scientific (Rochester, NY, USA). The NMR tubes (3 mm) were acquired from Bruker Ltd. (Milton, ON, Canada). Milli-Q water was used for all dilutions and stock solution preparations.

### 2.2. Urine and Serum Sample Collection

Mid-stream urine samples were collected from 20 healthy male and female volunteers of diverse ages, dietary preferences, and ethnic backgrounds (60% males, aged 22–41 years, mean 35.4 years of 10% African, 20% each of Asian—Chinese, Caucasian, and Middle Eastern, and 30% East Indian ethnicity [40]). Based on various features (color, creatinine levels, etc.), these urine samples also exhibited a broad range of hydration levels. A local pooled urine (L-PU) sample was prepared from these samples as previously described [41]. Commercial pooled urine (C-PU; 12 donors; 2 L) purchased from Lee BioSolutions (Maryland Heights, MO, USA) was prepared as previously described [42]. Prior to testing, the L-PU, C-PU, and normal urine samples were thawed on ice, vortexed, and then centrifuged to pellet any precipitated material.

Human urine samples were collected as part of a prospective, observational study designed to develop a screening test using metabolomics to discriminate between those at higher risk for CRC from other non-CRC conditions, as reported in our previous publication [30].

Human serum samples were collected from healthy volunteers. Serum was prepared using standard methods [43] by allowing drawn blood to clot for 30 min at room temperature (RT), followed by centrifugation at 1500–2000× *g* for 15 min and removal of the clear serum from the cell pellet. A standardized commercial pooled plasma sample (SRM 1950) was purchased from the National Institute of Standards and Technology (NIST) at Gaithersburg MD. All the serum/plasma samples were stored in −80 °C and thawed on ice before use. For all urine and serum samples collected from human participants, see the Institutional Review Board Statement (regarding ethics approvals) and the Informed Consent Statement at the end of the manuscript.

### 2.3. General Methodological Outline

**Figure 1:**
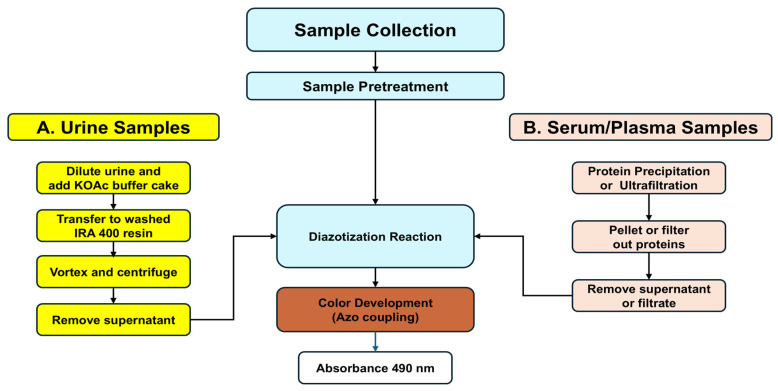
Flowchart showing the details of sample collection, pretreatment, and diazotization reaction followed by color development via azo coupling. Samples (spot urine or serum or plasma) are first collected and then pretreated. Urine samples are buffered using potassium acetate (KOAc) buffer cakes and interfering compounds are removed via treatment with IRA 400, an anion exchange resin. Serum or plasma proteins are removed via precipitation or ultrafiltration. The kynurenine in the supernatants from the pretreated urine or serum/plasma is then reacted via diazotization and the color produced via azo coupling.

For urinalysis, the colorimetric L-Kyn assay requires pretreatment of urine with a strong anion exchange resin (Amberlite^®^ IRA 400, Sigma-Aldrich, Oakville, ON, Canada) to remove interfering metabolites. This requires acidification of the sample with potassium acetate (KOAc) prior to being mixed with the IRA 400 resin. To detect L-Kyn in serum or plasma, proteins must be precipitated via acidic treatment with an organic acid (*p*-TsOH). After each biofluid has been pretreated, it is then acidified and reacted with diazotization reagents (Figure 1). Additional methodological details are provided below and in the Supplementary Materials.

#### 2.3.1. Preparation of Solid Potassium Acetate Buffer Cakes

Diazotization reactions typically require acidic pH levels to produce colored reaction products. As the pH of normal urine is 5.6–6.5 [44], we needed to lower the pH of the urine samples to allow the diazotization reaction to proceed and detect the presence of L-Kyn. This was achieved by adding lyophilized KOAc to the urine prior to passing the sample through the Amberlite^®^ IRA 400 resin. The KOAc (pH 5) was prepared from two solutions: glacial acetic acid and KOAc, which were mixed together and the pH adjusted to pH 5. This buffer solution was aliquoted into microfuge tubes, frozen, and lyophilized to create the KOAc buffer cakes. See the Supplementary Materials for details.

#### 2.3.2. Preparation of Amberlite^®^ IRA 400 Anionic Exchanger Resin

The Amberlite^®^ IRA (400/402) anionic exchanger resin was prepared in small volumes as previously described [40]. The anionic exchange resin was used so that interfering metabolites from small volumes of urine could be removed while maintaining measurable levels of L-Kyn. Then, 50 mg of washed resin was added to 1.5 mL microfuge tubes and stored at RT until used.

#### 2.3.3. Removal of Interfering Metabolites from Urine Samples

Urine samples, with or without spiked L-Kyn, were diluted 3×, added to one lyophilized KOAc buffer cake, and then transferred to microfuge tubes containing the washed IRA 400 anion exchanger resin. The urine samples were vortexed, the resin was centrifuged, and supernatant (containing L-Kyn) was aliquoted into PCR tubes. See the Supplementary Materials for details.

#### 2.3.4. Removal of Protein from Serum/Plasma Samples by Ultrafiltration or Precipitation

Before serum or plasma can be assayed for the presence of L-Kyn, abundant proteins such as albumin and lipoproteins [45], which can interfere with the assay, must be removed by one of two common methods: ultrafiltration (using 3 kDa Amicon filters) or precipitation (using *p*-TsOH solution). See the Supplementary Materials for details.

#### 2.3.5. Kynurenine Assay for Urine or Serum/Plasma

Resin-treated urine samples or deproteinized serum/plasma, aliquoted into PCR tubes, were stored on ice. Then *p*-TsOH was added, followed by freshly prepared NaNO_2_. The reactants were mixed and incubated briefly at RT. Then a freshly prepared solution of 2-naphthol was added. Reaction mixtures were transferred to a 96-well plate and absorbance at 490 nm (A_490_) was measured using a BioTek^®^ Synergy HT UV-Vis spectrophotometer (Winooski, VT, USA). See the Supplementary Materials for details.

### 2.4. Inhibition of Kynurenine Assay by Abundant Urinary Metabolites

To determine which abundant urinary metabolites interfered with the L-Kyn assay, low, medium, and high concentrations (based on the expected normal urinary values reported in the Human Metabolome Database (HMDB) [46]) of the various classes of urinary metabolites were prepared (see Table S1), added to 100 µM L-Kyn, and assayed by the described L-Kyn assay. The chosen compounds were classified into three main groups: (1) amines and amino acids (containing NH/NH_2_ groups, e.g., histidine), (2) reducing agents and sugars (e.g., ascorbic acid, mannitol), (3) acids (containing a COOH group, e.g., hippuric acid, glycolic acid). The percentage difference was determined by subtracting the A_490_ obtained for 100 µM L-Kyn from the A_490_ of 100 µM L-Kyn spiked with different concentrations of each metabolite, dividing by the A_490_ obtained for 100 µM L-Kyn, and then multiplying by 100 (see Equation (1)).

(1) $$\%\;difference=\frac{\left(A_{490}\;x\;µM\;Metabolite+100\;µM\;L-Kyn)-(A_{490}\;100\;µM\;L-Kyn\right)}{A_{490}\;100\;µM\;L-Kyn}\;x\;100\;$$

### 2.5. Preparation of Kynurenine Calibration Curves in Urine or Serum/Plasma

For both L-Kyn calibration curves prepared in urine or serum/plasma, a 10 mM L-Kyn stock solution was used. The 10 mM L-Kyn stock solution was prepared by diluting 100 μL of 100 mM L-Kyn (20.82 mg L-Kyn/1 mL 0.5 M HCl) with 900 μL of Milli-Q water. Both the 10 mM and 100 mM L-Kyn stock solutions were stored at −20 °C. The urine calibration curve (prepared in triplicate) used 200 μL of the 3× diluted C-PU aliquoted into PCR tubes and involved removing 0.5–6 μL of the diluted urine and replacing it with the same volume of 10 mM L-Kyn, generating solutions with 0–300 µM L-Kyn (see Supplementary Materials for details). These solutions were treated with IRA 400 resin and assayed by the L-Kyn assay. The A_490_ averaged values were plotted against the spiked-in concentrations of L-Kyn to generate a linear calibration curve. The standard deviation (SD), the coefficient of determination (R^2^), and the coefficient of variation (% CV, equal to the SD of each absorbance measurement divided by the mean, then multiplied by 100) were calculated using Microsoft Excel. The lower limit of detection (LLOD) and limit of quantification (LOQ) were derived from the measured A_490_ values from the unspiked blank (100 μL of buffered, unspiked, 3× diluted, and resin-treated PU and assayed via the L-Kyn assay). The LLOD was equal to 3 times the SD of this blank divided by the slope (see Equation (2)) while the LOQ was equal to 10 times the SD of the blank divided by the slope (see Equation (3)) [47].

(2) $$LLOD=3\times \frac{SD}{slope}$$

(3) $$LOQ=10\times \frac{SD}{slope}$$

The calibration curve prepared in pooled plasma used 100 μL of the deproteinized SRM 1950 aliquoted into PCR tubes and involved removing 0.125–1.25 μL of plasma and replacing it with the same volume of 10 mM L-Kyn, generating 12.5–125 µM L-Kyn (see Supplementary Materials for details). These solutions were assayed by the L-Kyn assay. The calibration curve was generated and the LLOD and LOQ were calculated by blank determination of 100 μL of the deproteinized, unspiked serum sample assayed via the L-Kyn assay and calculated as described for urine (see Equations (2) and (3)). The calibration curve for deproteinized serum was assumed to be identical to deproteinized plasma.

### 2.6. Preparation of Kynurenine Correlation Curves in Urine or Serum

To validate the assay performance in urine, 20 different urine samples from healthy Canadian volunteers were spiked with up to 300 μM L-Kyn and 22 healthy Nigerian samples were spiked with up to 140 μM L-Kyn to generate 52 and 44 different samples of each group (Canadian and Nigerian), respectively. Each spiked urine sample was pretreated with IRA 400 resin and assayed by the L-Kyn assay. An additional 13 unspiked urine samples from Nigerian CRC participants, pretreated and cleaned with IRA 400 resin, were assayed for their endogenous levels of L-Kyn. To validate the assay performance in serum, six human deproteinized serum samples were spiked with different amounts of L-Kyn (from 12.5 μM up to 125 μM). Forty different serum samples were generated and assayed by the L-Kyn assay. The concentrations of L-Kyn in the spiked samples (urine and serum) were determined from the spectrophotometric absorbances entered in the equation of linear regression generated from the L-Kyn calibration curves created using the C-PU and pooled plasma samples, respectively. The corresponding correlation graphs were generated by plotting the assay concentrations vs. theoretical (spiked) for the urine and the serum samples. For the 13 Nigerian CRC urine samples, assay vs. LC-MS concentrations were plotted. The R^2^ and the Pearson correlation coefficient were calculated using Microsoft Excel. For the Bland–Altman analysis, the means, differences, and SD of differences between the LC-MS/MS values and the colorimetric-assay-determined concentrations were also calculated using Microsoft Excel for Microsoft 365 MSO (Version 2505 Build 16.0.18827.20092).

### 2.7. Quantification of Kynurenine and Other Compounds by NMR Spectroscopy

Spiked urine samples were analyzed for L-Kyn, hippuric acid, histidine, mannitol, and tartaric acid concentrations using previously described NMR methods [41]. Aliquots (200 μL) of volunteer urine before and after treatment with the anionic exchanger resin IRA 400 were mixed with 5 × NMR buffer (50 μL; containing 750 mM potassium phosphate, pH 7.0, 5.00 mM deuterated 2,2-dimethyl-2-silapentane-5 sulfonate (DSS-d_6_), 5.84 mM 2-chloropyrimidine-5-carboxylic acid, and D_2_O 54% *v*/*v* in H_2_O), vortexed and centrifuged, and then loaded into 3 mm NMR tubes. NMR spectra were recorded on a 700 MHz Avance III HD Bruker (Bruker Biospin, Rheinstetten, Germany) NMR spectrometer equipped with a cryogenically cooled triple resonance probe (TCI), as previously described [41]. All spectra were processed using the TopSpin software (version 3.6.2) and L-Kyn, hippuric acid, histidine, mannitol, and tartaric acid in the urine samples were identified and quantified using Chenomx NMR Suite software (version 8) as previously described [41]. Each metabolite concentration was corrected for dilution with the 5 × NMR buffer by dividing by 0.8. The percentage difference in concentration before and after treatment with the IRA 400 resin was calculated by subtracting the concentration of the metabolite (in µM) after treatment from the concentration of the metabolite (in µM) before treatment, then dividing by the concentration of the metabolite (in µM) before treatment and finally multiplying by 100 (see Equation (4)).

(4) $$\%\;difference=\frac{\left(µM\;metabolite\;before\;treatment-µM\;metabolite\;after\;treatment\right)}{µM\;metabolite\;before\;treatment}\times 100$$

### 2.8. Quantification of Kynurenine by Liquid Chromatography Tandem Mass Spectrometry

A targeted, quantitative, MS-based metabolite assay was used to detect and quantify 142 urinary compounds, including amino acids and derivatives (such as L-Kyn), biogenic amines, glucose, and organic acids (especially uric acid) using a combination of reversed phase liquid chromatography (LC) and direct flow injection (DFI) with MS/MS (LC/DFI MS/MS). Details about the method, derivatization strategy, separation protocol, MS methods, calibration, and metabolite quantification process have been previously described [48].

## 3. Results

### 3.1. Chemistry of Diazotization and Coupling Reaction to Detect Kynurenine

**Figure 2:**
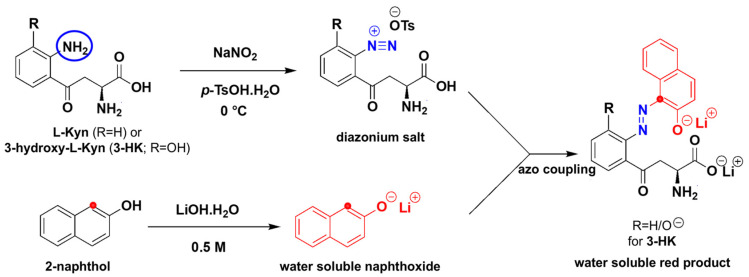
Chemistry of diazotization reaction of the aromatic amine of kynurenine (L-Kyn) followed by azo coupling. The top left reaction shows the diazotization of L-Kyn (colored black) using *p*-TsOH (replacing the commonly used HCl) and NaNO_2_, reacted on ice. The blue encircled NH_2_ group is diazotized by the electrophile NO^+^ (generated when NaNO_2_ is mixed with *p*-TsOH), producing the diazonium salt intermediate. The resulting diazonium salt (diazo site is colored blue) acts as an electrophile when reacted with the electron-rich naphthoxide (colored red and the electron-rich 2-position is highlighted by a red dot; prepared by dissolving 2-naphthol with a base, 0.5 M LiOH or NaOH), leading to the formation of a highly conjugated, intense red-colored water-soluble azo dye (azo-coupling step). Note: this reaction may occur with other R-substituted L-Kyn such as 3-hydroxykynurenine (3-HK). R = H for L-Kyn; R = OH for 3-HK. Abbreviations: LiOH—lithium hydroxide; NaNO_2_—sodium nitrite; -OTs—tosylates; *p*-TsOH—*para*-toluenesulfonic acid.

To the best of our knowledge, the diazotization reaction has not been applied to L-Kyn. Diazotization [49], shown in Figure 2, commonly uses HCl as a source of acid. A mixture of NaNO_2_/HCl generates the effective electrophile NO^+^ which diazotizes the aromatic amine of L-Kyn at 0 °C, resulting in the diazonium salt. In this process, the aromatic NH_2_ acts as a nucleophile. The resulting diazonium salt acts as an electrophile when reacting with the electron-rich 2-naphthol, resulting in a highly conjugated azo dye with a bright red color. Aliphatic amines can also undergo the same reaction but do not contribute to the color formation due to the lack of extended conjugation. We made two strategic changes to this traditional diazotization protocol. First, we replaced the liquid HCl with solid *p*-TsOH [50] for easier handling and to develop a more portable, more easily shipped, user-friendly solid assay. Second, we used LiOH/NaOH to deprotonate the final product (both the phenolic OH and COOH groups) which not only enhanced the color intensity through extended conjugation but also transformed the colored product into a water-soluble salt, ensuring consistent absorbance readings.

### 3.2. Optimization of the Assay Conditions in Urine and Serum

We optimized this diazotization reaction to work with complex biofluids (various urine and serum/plasma samples) and in much smaller volumes with the goal of creating a cost-effective user-friendly assay. We first optimized the reaction to work with urine. The diazotization reaction requires strongly acidic conditions to acidify any aromatic amine. Our initial protocol used HCl to acidify and protonate L-Kyn followed by addition of NaNO_2_, to generate the diazonium salt of L-Kyn followed by coupling with 2-naphthol, prepared in EtOH. However, this yielded a poor color gradient and only worked with urine samples from well-hydrated individuals. In addition to HCl not working optimally with urine, it is also highly corrosive and releases irritating fumes, making it unsuitable for a portable, shippable, easily stored or automated, user-friendly assay. We searched for safer alternatives and tried *p*-TsOH, which does not release volatile fumes. We found that a small amount of *p*-TsOH (10 μL of 0.5 M *p*-TsOH) consistently lowered the pH of 50 different urine samples to 1 and served as an excellent replacement for HCl in our assays. Although *p*-TsOH lowered the pH of numerous individual urine samples, we used C-PU to further optimize the assay conditions due to the large volumes available for testing. It is important to note that NaNO_2_ reacts with any NH_2_ group containing metabolites (such as amino acids, indoles—bicyclic compounds with a ring nitrogen—etc.), including urea. Given that normal urinary levels of urea are up to 20,000 times greater than those of L-Kyn [31], an excess of NaNO_2_ was required to ensure that NaNO_2_ was not consumed prior to its reaction with L-Kyn. After spiking 50 different urine samples covering a range of natural dilution/hydration levels with very high concentrations of L-Kyn (500 μM), we found 10 μL of aq. NaNO_2_ yielded the best results by producing the most intense color and maximal absorbance.

The amount of 2-naphthol was also optimized since it can potentially react with any electrophilic compounds present in urine. We determined that excess naphthol (64.7 mM) also needed to be added. Initially, we used EtOH to dissolve 2-naphthol since it is insoluble in water (or urine). However, color formation, particularly for urine samples from dehydrated individuals (expected to have higher concentrations of L-Kyn), was faint. We attempted to improve color formation by replacing 2-naphthol with other conjugated aromatic nucleophiles such as 9-phenanthrol, which led to a yellow product and 8-hydroxyquinoline, where no reaction was observed. To replace EtOH as the solvent, we tried water-soluble versions of 2-naphthol such as 5-hydroxynaphthalene-1-sulfonic acid, which did not react, naphthalene-1,5-diol, which had faint color formation with some precipitation, and naphthalene-2,6-diol, which showed intense color formation only when EtOH was added (which we were trying to avoid). Thus, no other conjugated nucleophile improved color formation, suggesting that 2-naphthol was the best choice. We then replaced EtOH with aqueous LiOH/NaOH, which had multiple advantages. 2-Naphthol dissolves in aqueous LiOH/NaOH and the resulting naphthoxide is a strong nucleophile which reacts instantly with the diazonium salt. This leads to a highly conjugated colored product. The product is also water-soluble as it exists as a naphthoxide/carboxylate salt under basic conditions. Moreover, the color intensity of the product is enhanced under basic conditions because the naphthoxide anion of the product participates in an extended conjugation. When the 2-naphthol (dissolved in LiOH/NaOH) reacted with the L-Kyn diazonium salt, an excellent color gradient was seen with diverse urine samples. The amount of base was also adjusted so that the colored product was soluble in water without forming precipitates (which are challenging to quantify spectrophotometrically) while avoiding excess base which caused the reaction mixture to turn yellow (not red). When 2-naphthol was prepared in base (0.5 M of either LiOH or NaOH), the best results were obtained, and the product was stable and soluble for >30 min after being formed. When the colored products were scanned from 200 to 600 nm, the absorption maximum was 490 nm when it was prepared in base or EtOH. However, the measured absorbance using base was twice that measured with EtOH, allowing us to obtain lower LLODs.

To optimize the reaction to work with serum/plasma, a deproteinization step was required since the abundant proteins in serum/plasma (albumin and lipoproteins [45]) contain aromatic amines and would yield a high background reaction with the diazotization reaction. Ultrafiltration using a 3 kDa filter was effective in removing proteins from serum and the filtrate reacted well with the *p*-TsOH, NaNO_2_, and the basic solution of 2-naphthol. However, since these protein filters are expensive, and serum samples are manually intensive to prepare using these filters, they would not be ideal for a cost-effective, user-friendly assay. Therefore, we decided to use chemical precipitation instead. Although protein precipitation can be achieved with acids or alcohols, we selected an acid since the first step of diazotization requires strongly acidic conditions. We first tried trichloroacetic acid which caused a very intense yellow background when the L-Kyn assay was performed. We then used 2 M *p*-TsOH solution to precipitate protein. After the precipitated proteins were pelleted by centrifugation, complete protein removal from the supernatant was confirmed by a standard Bradford protein assay. In the final serum/plasma deproteinization step, 4× less *p*-TsOH (0.5 M) was used, which also corresponded to the optimized concentration to assay L-Kyn in urine. Because *p*-TsOH was used for protein precipitation, no additional *p*-TsOH was required to perform the diazotization step in L-Kyn assay.

### 3.3. Sample to Sample Variation and Potential Interfering Metabolites

In contrast with enzymatic reactions which bind substrates with high avidity, chemical assays tend to be less specific since the reactions may occur with a wide range of other reactants having the same functional group(s). Since human urine consists of several thousand metabolites [31] from diverse chemical classes, it was expected that some common urinary compounds may interfere with this L-Kyn assay by reacting with the diazotization reagents, thereby enhancing or preventing color formation. When we applied our optimized assay to multiple healthy urine samples spiked with various concentrations of L-Kyn, high sample to sample variation was observed (Figure S1), particularly for concentrations ≤100 µM L-Kyn. Unspiked urine samples also showed large variations in absorbance. This suggested that other metabolites in urine were interfering with the L-Kyn assay. To identify which compounds might be causing this, we systematically added low, medium, and high concentrations of each of 21 of the most abundant urinary metabolites (Table S1) to an aqueous sample containing 100 µM L-Kyn. We then conducted the L-Kyn assay and calculated the percentage difference in the observed color change to determine which metabolites interfered with the assay. The high-abundance compounds were classified into three main groups: (1) amines and amino acids (containing NH or NH_2_ groups, e.g., urea, histidine), (2) reducing agents and sugars (e.g., ascorbic acid, mannitol), and (3) acids (containing a COOH group, e.g., acetoacetic acid, glycolic acid, hippuric acid). The concentrations of the potential interfering compounds added to 100 µM L-Kyn were based on the expected normal urinary values reported in the HMDB [46]. Representative graphs showing the percentage difference in absorbance measurements when interfering metabolites were added, compared to 100 µM L-Kyn alone, are shown in Figure S2.

The greatest decreases in absorbance were seen when 100 µM L-Kyn was spiked with ascorbic acid (100, 300, and 600 µM), a strong reducing agent, followed by hippuric acid (500, 1000, and 1500 µM), mannitol (100, 300, and 600 µM), and to a lesser extent by acetoacetate (100 µM), glycolic acid (50 µM), and tartaric acid (25 µM). The greatest increases in absorbance were seen with glycolic acid (150 and 500 µM), histidine (250, 500, and 1000 µM), and glutamine (250, 500, and 1000 µM) and, to a lesser extent, acetoacetic acid (200 µM) and tartaric acid (50 µM). In addition to urea, and the indole compounds mentioned earlier, these interfering metabolites could be contributing to the variable absorbances seen in the various healthy volunteer urine samples.

### 3.4. Urine Clean-Up with Amberlite IRA 400

We have previously developed other colorimetric assays to detect urinary hippuric acid [41] and diacetylspermine or DAS [40]. With both assays, we also encountered similar issues with interfering urinary metabolites. For hippuric acid and DAS, we successfully used cation and anion ion-exchange resins [40] and/or urinary dilution [41] to overcome those chemical interference issues. In the case of DAS, we implemented minimal sample preparation steps by developing a simple two-column system that both removed interfering metabolites and concentrated DAS, which was subsequently adapted for use with our POC colorimetric biosensor. Building on this approach, we applied similar methods for improving the performance of this assay. Various chemical neutralization agents (to eliminate or remove reactive amines and organic acids) were initially tried including charcoal, bentonite, and kaolinite. Charcoal completely removed L-Kyn along with interfering metabolites. Bentonite and kaolinite removed 30–40% L-Kyn without removing other interfering compounds. We then tried strong anionic and cationic exchanger resins to remove the interfering metabolites while retaining L-Kyn in the flow through. L-Kyn is an amino acid with both carboxyl (COOH) and amino (NH_2_) side groups and has an isoelectric point of pI 6.11 (net neutral change). It would not be expected to bind to an anionic exchange resin (which binds negatively charged compounds) if it was protonated (positively charged) if dissolved in an acidic solution. As its optimal buffering range is from pH 3.6–5.6, we selected acetate to uniformly lower the pH of urine samples (and L-Kyn) below the isoelectric point of L-Kyn. We assessed the binding at several pHs levels below 6 and, after extensive NMR studies performed on urine samples treated with IRA 400, pH 5 was selected as the optimum pH. We also used *p*-TsOH to lower the pH to 1 and then performed the resin treatment. This very low pH led to too much L-Kyn binding to the resin and thus it was abandoned. We tried other anion exchange resins (e.g., IRA 67, Dowex 1 × 8) but did not achieve the desired color intensity. We also used cation exchange resins (e.g., Dowex 50WX8-100, Amberlite CG-120) [40] to trap L-Kyn, followed by elution with a high-salt solution, but the assay produced yellowish brown precipitates instead of the desired, water-soluble, deep red dye.

We also optimized the amount of acetate buffer that should be added to urine. Using 50 different urine samples with different concentrations and volumes of acetate buffer, we determined that 200 μL of a urine sample should be added to 250 μL of 0.1 M KOAc buffer (pH 5) to ensure all urine samples would be at pH 5. We then added Amberlite IRA 400 (50 mg) to the 200 μL of acetate-buffered urine and assayed the metabolites in the supernatant by NMR to determine if any interfering metabolites were bound to the IRA 400 resin and if any were removed. An ^1^H-NMR spectrum of C-PU before and after treatment with IRA 400 shows that hippuric acid was removed by the resin (Figure S3). The abundance of any potential interfering metabolites was quantified by NMR, both before and after IRA 400 treatment, and the percentage difference before and after treatment was calculated (Table 1). NMR was chosen to quantify the interfering metabolites because, unlike LC-MS/MS—which requires extensive sample preparation, isotopic standards, chromatographic separation, and complex analysis—NMR offers simpler sample preparation, comprehensive detection, and more reliable quantification of water-soluble metabolites. Here, 96.5% tartaric acid, 95.8% hippuric acid, 38.3% mannitol, and 6% histidine were removed in the IRA 400-treated urine. Previous studies in our laboratory also demonstrated that the IRA 400 resin removed 91.3% hippuric acid, 100% indole acetic acid, 100% ascorbic acid, and 99.7% uric acid and urobilin [40], showing that this resin is effective in removing interfering agents from urine. While the concentrations of the interfering compounds were substantially reduced after treatment with this strong anion exchanger, about 13.9 ± 5.6% L-Kyn (attributed to its deprotonated COOH group at pH 5) was also removed by the IRA 400 treatment when eight different Canadian volunteer urines were spiked with 0–300 µM L-Kyn (Figure S4a). Only one sample showed a higher percentage difference (D4 at 23%; Figure S4b). Overall, the percentage of removal of L-Kyn by the IRA 400 resin was quite consistent and relatively low across the tested urine samples, falling well within the expected variability of a quantitative assay.

### 3.5. Finalized Protocol for Urine and Performance Assessment

When sample to sample variation was tested after IRA 400 resin treatment using 10 different urine samples, we found that the previously observed absorbance variations were significantly reduced. When we performed a correlation study spiking only hydrated urine samples with 0–200 μM L-Kyn, we obtained an R^2^ = 0.92. When dehydrated urine samples were included, the R^2^ dropped to 0.87, suggesting that other metabolites, with higher concentrations in the dehydrated urine samples, were still interfering with the assay. To further minimize the chemical interference, we diluted the IRA-400-filtered urine samples three times (3×). Using 3×-diluted IRA-400-treated samples, very little variation in the unspiked samples was observed with urine samples from both well-hydrated and dehydrated individuals.

**Figure 3:**
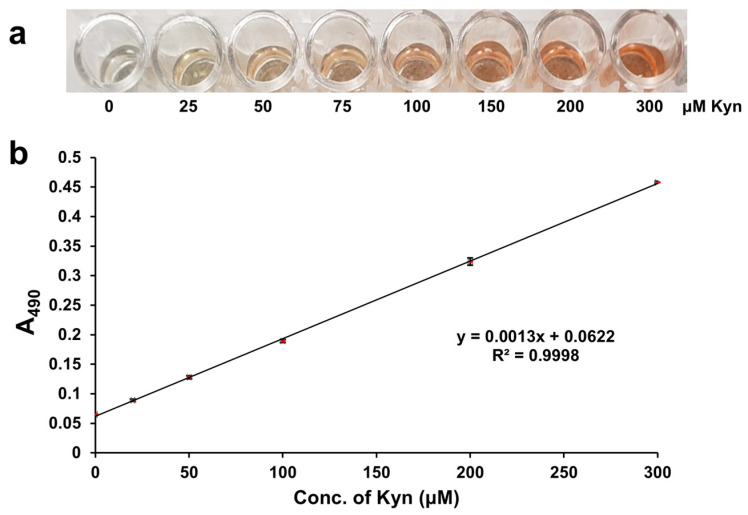
Kynurenine (L-Kyn) assay performed with urine. (**a**) Color gradient of 3× diluted urine spiked with up to 300 μM L-Kyn, then filtered with the anionic exchanger resin IRA 400 to remove interfering metabolites before detection with the L-Kyn assay. (**b**) Calibration curve generated when 3× diluted commercial pooled urine was spiked with up to 300 μM L-Kyn, treated with IRA 400 resin, and then reacted via the L-Kyn assay.

When 0–300 µM L-Kyn was spiked into 3X diluted urine, then filtered with the anionic exchanger IRA 400 and reacted via the L-Kyn assay, a clear yellow to dark red color gradient was observed (Figure 3a). When the A_490_ of these reactions was plotted against the concentration of L-Kyn spiked into the diluted C-PU, a linear calibration curve with R^2^ = 0.9998 was generated (Figure 3b). Subtracting the background absorbance of unspiked samples yielded a linear calibration curve with the same R^2^ (0.9998; see Figure S4c). When 0–300 µM L-Kyn was spiked into 3X diluted Canadian urine samples and treated with resin, levels determined from calibration curves with and without background subtraction were almost identical (see Figure S4d). These results demonstrated that 3× sample dilution followed by IRA 400 pretreatment effectively removed almost all background interference and sample to sample variations. Therefore, background subtraction was not required for data generation.

When performed in triplicate, low coefficients of variation (CVs) were calculated (ranging from 0.4–2.1%; Table 2) with LLOD = 1.34 μM and LOQ = 4.46 μM. Notably, LLODs and LOQs could not be calculated using background-subtracted unspiked blanks, as the blank values were zero. The high consistency of the assay, reflected in low CV% and SDs (Table 2), underscores its reliability for potential clinical applications.

**Figure 4:**
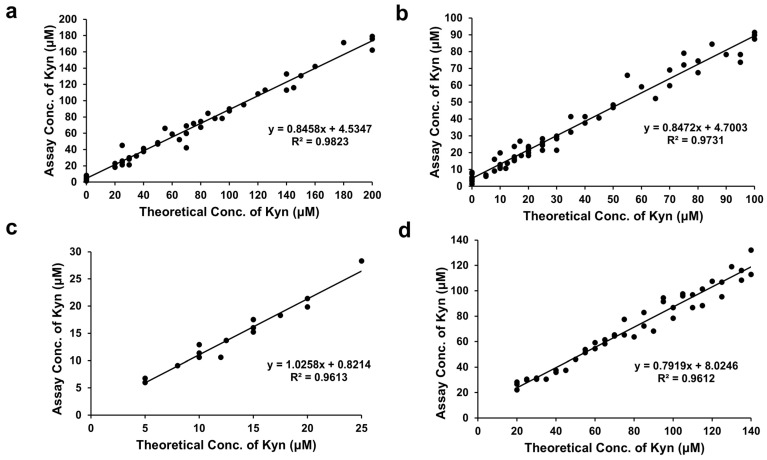
Correlation curves of assayed concentrations versus theoretical concentrations of kynurenine (L-Kyn) spiked into diluted urine, treated with an anionic exchanger resin and processed via the L-Kyn assay. Correlation between assay and theoretical concentrations of 3× diluted urine samples from healthy Canadian volunteers randomly spiked with (**a**) up to 200 μM L-Kyn, (**b**) up to 100 μM L-Kyn, and (**c**) from 5–25 μM L-Kyn before pretreatment with IRA 400 and L-Kyn assay. (**d**) Correlation between assay and theoretical concentrations of up to 140 μM L-Kyn randomly spiked into urine samples from healthy Nigerian volunteers.

The assay performance was validated by conducting a correlation study with 20 healthy Canadian samples spiked with increasing levels of L-Kyn. The R^2^ values were 0.98 (0–200 μM spiked L-Kyn), 0.97 (0–100 μM spiked L-Kyn), and 0.96 (5–25 μM spiked L-Kyn) and the slopes were 0.85, 0.85, and 1.03, respectively (Figure 4a–c). Notably, the assay’s excellent linearity (R^2^ = 0.96), even at low L-Kyn concentrations (5–25 µM), suggests its utility in detecting subtle changes in L-Kyn levels, which could be crucial for early disease detection or monitoring treatment responses. These performance studies demonstrate that the L-Kyn assay can detect a little as 5 µM and as much as 300 µM L-Kyn when assayed on 3×-diluted IRA-400-treated urine.

**Figure 5:**
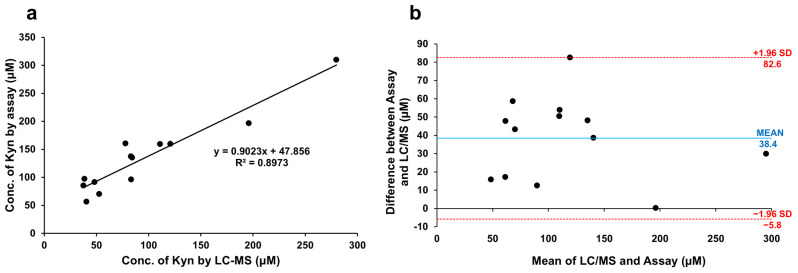
Comparison between kynurenine (L-Kyn) assay and liquid chromatography–mass spectrometry (LC-MS) in quantifying L-Kyn. Thirteen urine samples from patients from the Nigerian colorectal cancer study were evaluated by each method. (**a**) Linear correlation between the LC-MS and the colorimetric L-Kyn assay. (**b**) Bland–Altman plot showing mean bias and upper and lower 1.96 SD levels of agreement.

Previous LC-MS studies in our laboratory determined that urine samples from Nigerian participants contained from 0 to 300 μM L-Kyn (Figure S5; [30]). Diluted 3X, we expect these urine samples to contain up to 100 μM L-Kyn. We conducted a correlation study spiking 22 healthy Nigerian samples with slightly more L-Kyn than expected (0–140 μM), achieving an R^2^ = 0.96 and a slope of 0.79 (Figure 4d). Finally, we quantified L-Kyn in 13 (non-spiked) Nigerian urine samples from participants in the CRC study by LC-MS and by our colorimetric assay, obtaining an R^2^ = 0.90, slope = 0.90, and Pearson coefficient = 0.95 (Figure 5a). Using the Bland–Altman plot (Figure 5b), the mean bias was 38.4 μM with almost all measurements falling within the 1.96 SD limits of agreement (−5.8 to 82.6 μM). Most L-Kyn assay levels were higher than LC-MS-determined concentrations (see Table S2). The positive Pearson correlation, high R^2^, and slope between our colorimetric assay and LC-MS measurements using Nigerian clinical CRC samples suggest a strong linear correlation between the methods. However, as the L-Kyn assay overestimates compared to LC-MS, further optimization may be required to improve the agreement between the two methods. Despite this bias, the assay demonstrates potential as a practical and cost-effective alternative to LC-MS for L-Kyn quantification.

### 3.6. Assay Performance with Serum/Plasma

**Figure 6:**
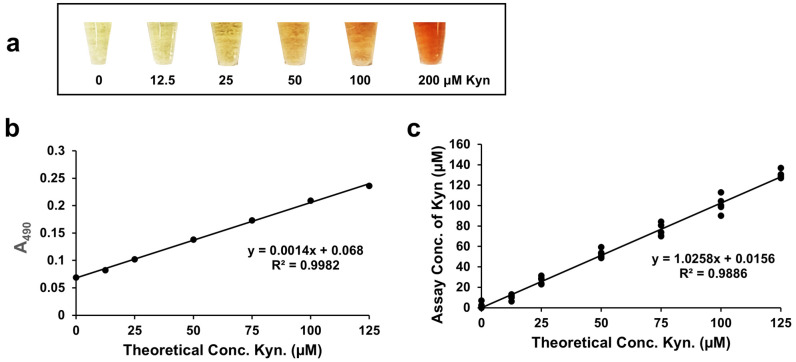
Serum/plasma deproteinized with para-toluenesulfonic acid (*p*-TsOH), spiked with kynurenine (L-Kyn), and processed using the L-Kyn colorimetric assay. (**a**) Color gradient of deproteinized serum spiked with 12.5–200 µM L-Kyn; (**b**) Calibration curve generated when deproteinized pooled serum was spiked with 0–125 µM L-Kyn; (**c**) Correlation of assayed versus theoretical concentrations of 40 different deproteinized serum samples spiked with 0–125 µM L-Kyn.

L-Kyn levels in plasma and serum are also diagnostically important [13,14,15,18,19,20,27,50,51] and for this reason we also adapted our L-Kyn assay to work with deproteinized serum/plasma. After precipitating protein from serum using *p*-TsOH, spiking the supernatant with 0–200 µM L-Kyn, and conducting the colorimetric L-Kyn assay, yellow to intensely red-colored products were clearly produced (Figure 6a). While the background absorbance at 490 nm was a little higher with serum/plasma compared to urine, no background absorbance was subtracted from the spiked readings since the absorbance was quite low. When deproteinized serum/plasma was spiked with 0–140 µM L-Kyn and the L-Kyn assay performed, a calibration curve with a high R^2^ (0.9982) was produced (Figure 6b) with an LLOD = 1.24 μM and an LOQ = 4.12 μM. When deproteinized serum samples from healthy volunteers were spiked with 0–125 μM L-Kyn and quantified by the L-Kyn assay (Figure 6c), again a high R^2^ (0.99) and slope (1.03) were seen. This suggests that the optimized L-Kyn assay has good linearity and good accuracy in detecting L-Kyn in deproteinized serum/plasma samples across physiologically relevant concentration ranges.

## 4. Discussion

Diazotization is a well-known chemical reaction [49] for transforming aromatic amines into diazonium salts, which are key intermediates used to synthesize intensely colored azo dyes (e.g., Sudan Red). In this paper, we used diazotization and azo coupling to develop a simple colorimetric assay that can detect and quantify L-Kyn, an aromatic amine, in human urine and serum/plasma samples. The diazotization reaction is a two-step process where L-Kyn is first acidified and converted to a diazonium salt and in the second step, the diazonium salt (acting as an electrophile) reacts with an electron-rich aromatic compound (2-naphtholoxide) to form a visible azo dye.

Key to successfully implementing this assay with biological samples was developing a simple “cleaning” step prior to running the reaction. For urine, the samples were diluted 3× and treated with a strong anionic exchanger resin (Amberlite IRA 400) to remove most of the interfering metabolites (such as ascorbate, hippuric acid, tartaric acid). For serum, the samples were deproteinized using *p*-TsOH to remove reactive amino acid side chains. We also showed that careful, systematic optimization of the assay, often involving relatively small changes in reagent and sample preparation, were able to yield significant boosts to the assay’s performance. Unlike previously published colorimetric L-Kyn assays, our method achieved reliable quantification at physiologically relevant concentrations in both urine and plasma/serum. Specifically, it achieved good linearity and accuracy with spiked urine samples (ranging from 5–300 µM) as well as unspiked clinical urine samples from Nigerian CRC study participants as verified by LC-MS/MS. Using deproteinated serum samples, we achieved similar results using the same assay configuration.

This optimized L-Kyn assay could provide a cost-effective alternative for clinical diagnostics and disease monitoring, particularly in low-resource settings. It represents an essential step in expanding the repertoire of chemical tests integrated into our POC colorimetric biosensor platform [39]. As with previously described assays for DAS [40] and creatinine [39], the incorporation of solid-phase reagents and simple column purification ensures compatibility with both manual and automated workflows.

### 4.1. Interfering Metabolites and Their Removal to Optimize Kynurenine Detection

Chemical assays are known to be less specific than enzymatic or antibody assays, largely because they rely on functional group reactivity, which can lead to unintended side reactions with structurally similar metabolites. Urine contains thousands of metabolites from diverse chemical classes, many of which may interfere with this assay by enhancing or inhibiting color formation. For the L-Kyn assay, we categorized the interfering metabolites into four distinct groups based on their likely mechanisms of interference: (1) compounds containing NH_2_ and NH (urea, indoles, any amino acids, proteins, and peptides) that compete with L-Kyn for diazotization. Adding an excess of sodium nitrite (NaNO_2_) ensured that all NH_2_- and NH-containing compounds, including L-Kyn and urea (the most abundant amine-containing metabolite in urine; [31]), were diazotized. (2) Any diazotized products from group 1 will react with naphthoxide to form colored side products. (3) Any reducing agents (ascorbate, polyols, alcohols, sugars) that destabilize the diazonium salt. (4) Other compounds, such as organic acids (electrophiles), colored compounds like urobilin, and any nucleophiles (such as urea, phosphates, sulfates), can potentially attack the diazonium salt of L-Kyn, preventing color formation. This is particularly true for unconjugated compounds. Adding an excess of naphthoxide, an extremely powerful nucleophile, outcompetes other nucleophiles present, causing instant color formation.

The removal of the interfering agents was crucial for creating an optimal L-Kyn assay. Amberlite^®^ IRA 400, a strong anion exchange resin, removed many negatively changed urinary interfering compounds while sparing L-Kyn. Sample dilution (3×) further improved performance and minimized sample to sample variation. For serum/plasma, deproteinization with *p*-TsOH effectively eliminated proteins and amino acids that could interfere through nucleophilic side chains or color-absorbing moieties.

In developing this assay, we prioritized methods and reagents that could be easily transported, stored for long periods of time, and adapted to robotic automation. All reagents were selected or formulated to be chemically stable in solid form for shipping and storage. In some cases, the reagent solutions were prepared and then dried on thin sheets of polydimethylsiloxane (PDMS). The availability of solid, stable reagents will be key for developing a portable, storable, easy-to-use test kit for L-Kyn detection in field settings or resource-limited environments, in combination with our POC metabolomic biosensor. This is particularly important where liquid handling and storage of reagent solutions may be challenging. While full robustness testing under field conditions was outside the scope of this work, the assay was deliberately designed to accommodate real-world variables including tolerance to extreme pH and reliance on dry reagents.

### 4.2. Kynurenine Detection in Serum

In our study, the development of the serum assay was straightforward following effective protein removal, as evidenced by the excellent correlation (R^2^ = 0.99) with our assay results. Protein removal was achieved using either 3 kDa filtration or *p*-TsOH, which also serves a dual purpose as a key reagent in the diazotization process. Serum/plasma levels of L-Kyn can serve as a useful biomarker for various conditions, including cancer [13,14], diabetes, and long COVID [51]. For instance, patients with long/post-COVID symptoms have demonstrated significantly higher serum L-Kyn levels, averaging 8.77 ± 1.72 µM, with a range of 5.5 to 16.6 µM [51]. These values are well within the LLOD and LOQ range our serum/plasma colorimetric assay and certainly highlight the potential of using this assay in clinical applications.

### 4.3. Comparison to Other Chemical Assays for Detecting and Quantifying Kynurenine

As noted earlier, there are several other colorimetric assays that have been described for detecting and quantifying kynurenine—although not necessarily in biofluids. One recently described method used 3-formyl-4-(ethylthio)-7-(diethylamino)-coumarin to detect clinically relevant ranges of L-Kyn with a very low LOD but in a chemically simple synthetic urine (Surine^TM^) [38]. Even though the reported range and LOD was better than our chemical assay, it is notable that the assay was not tested on real urine samples. Other amino-containing metabolites such as amino acids could react, yielding more variable results that were seen with Surine^TM^. Moreover, the coumarin compound is not commercially available, making further assessment difficult.

Other aldehyde-based colorimetric chemical assays can detect L-Kyn by forming a conjugated Schiff base upon reaction with the aromatic NH_2_ of L-Kyn such as 4-dimethylamino benzaldehyde (DMAB) or Ehrlich’s reagent. DMAB has been used to detect L-Kyn in cell culture supernatants [52,53]. When trichloracetic acid is added to culture supernatants, N-formyl-kynurenine is hydrolyzed to L-Kyn, which can be detected when DMAB is added [52]. However, this assay appears to work only for very chemically “clean” samples. When DMAB was added to IRA-400-treated urine, poor color changes were seen and only at concentrations exceeding 100 μM L-Kyn—far above physiological levels. Furthermore, DMAB is known to react with indoles [54], urea [36], and urobilinogen [37], abundant metabolites in urine and other biofluids. Similarly poor results were seen with another aldehyde, 4-dimethylaminocinnamaldehyde (DACA). This a reagent is commonly used to quantitatively detect urinary *p*-aminohippuric acid [55,56], a compound that is structurally similar to L-Kyn. However, when DACA was tried, a very intense red color formed regardless of the urinary concentration of L-Kyn. We also tried other conjugated aldehydes such as 4-nitrobenzaldehyde, pyridine-4-carboxaldehyde, and 4-(4-pyridyl)benzaldehyde, but none of them produced any colored products with urine. Taken together, these findings highlight the limitations of existing colorimetric L-Kyn assays and underscore the novelty and utility of our diazotization-based method. To the best of our knowledge, this novel diazotization assay appears to be the most sensitive and most reproducible colorimetric method for the detection and quantification of L-Kyn in biofluids.

## 5. Conclusions

In this study, we developed a novel diazotization-based colorimetric chemical assay to detect and quantify L-Kyn in human biofluid samples at physiologically relevant concentrations. This two-step reaction, which had not previously been applied to L-Kyn detection, uses simple, commercially available reagents to generate an intensely colored azo dye product, providing a rapid, sensitive, cost-effective alternative to conventional analytical methods. By pretreating urine with a strong anion exchange resin (IRA 400) to remove interfering metabolites or by precipitating serum/plasma proteins using acid (*p*-TsOH), we found that the assay could be optimized to give excellent linearity, accuracy, and reproducibility. Indeed, the optimized assay achieved good linearity and accuracy with urine samples from participants in the Nigerian CRC study when assay values were correlated with L-Kyn quantified by LC-MS. We also refined the assay to ensure that all key reagents could be inexpensively purchased, easily acquired, and shipped and stored as solids. We also designed the assay so that all sample preparation steps (cleaning, precipitation, measurement) could be performed with low-cost, easily available equipment with an eye towards robotic automation. Our results highlight the assay’s potential as a practical, low-cost alternative to existing analytical techniques for L-Kyn quantification, particularly in resource-limited settings.

Despite its promising performance in both human urine and serum samples, the assay has some limitations. One such consideration involves the approach used for calibration. While pooled biofluids (C-PU and pooled serum) were used for calibration due to their reproducibility and accessibility, we recognize that pooling may mask individual variability in matrix components such as proteins, electrolytes, and endogenous metabolites, potentially underrepresenting matrix effects observed in individual patient samples. Beyond these matrix-related limitations, chemical interference from specific endogenous compounds may also affect assay accuracy. The assay performs best with well-hydrated, normally colored urine samples as extremely dehydrated (deep yellow or orange) urine samples still have high amounts of urobilin, even after resin pretreatment. This residual urobilin can interfere with the color formation, generating false positive reactions, possibly contributing to the higher concentrations of L-Kyn quantified by the L-Kyn assay compared to LC-MS/MS, as observed in the Bland–Altman analysis. (Further dilution of these darkly colored samples (an additional 2×) may mitigate this interference). Similar discrepancies have been reported in studies comparing immunoassays with LC-MS [57].

Another possible source of interference is 3-hydroxykynurenine (3-HK), which may also react in the L-Kyn assay (see Figure 2). Reported urinary concentrations of 3-HK range from 1.0–3.4 µmol/mmol creatinine [46], suggesting that the measured L-Kyn levels in Nigerian CRC participant samples could reflect a combined signal from both L-Kyn and 3-HK. However, our LC-MS/MS assay did not target 3-HK, so its levels in these samples remain unknown. Further studies are needed to quantify urinary 3-HK and to confirm whether it contributes to color formation in this assay, which may help explain the bias observed in the Bland–Altman plot. Aromatic primary amines/anilines with available NH or NH_2_ moieties can also potentially interfere by also generating diazonium salts that react with the 2-naphtholoxide, producing color.

Detection ranges in serum are still higher than we would ideally like to see and could benefit from further refinement to remove the background color or to concentrate L-Kyn. Further validation across larger and more diverse patient populations is necessary to fully assess the assay’s clinical applicability and robustness. Future work should explore the integration of this method into robotic, POC diagnostic devices such as our metabolomic biosensor [39] and evaluate its performance in detecting L-Kyn across a broader range of L-Kyn-related pathologies. This work lays the foundation for integrating the assay into our automated POC diagnostic platform [39], which we have been successfully used to implement many other colorimetric metabolite tests. By fully characterizing the chemistry, validating performance, and minimizing required sample preparation, we have ensured that this L-Kyn assay is ready for the next phase of integration into our biosensor platform.

## Figures and Tables

**Table 1:** Nuclear magnetic resonance (^1^H-NMR) levels of metabolites in urine sample B4 before and after treatment with IRA 400, an anionic exchanger resin (*n* = 1).

Treatment	L-Kyn	Hippuric Acid	Mannitol	Histidine	Tartaric Acid
B4 untreated (μM)	185	1234	120	235	520
B4 treated (μM)	124	52.4	74	221	18
Percentage difference	33	95.8	38.3	6	96.5

**Table 2:** Standard deviations and coefficient of variance (CV %) for the kynurenine calibration curve prepared in IRA-400-pretreated urine (in triplicate).

Conc. of Kyn (μM)	0	20	50	100	200	300
Average	0.065	0.089	0.128	0.189	0.324	0.458
Standard Deviation	0.00058	0.00173	0.00265	0.00252	0.00624	0.00173
CV (%)	0.9	1.9	2.1	1.3	1.9	0.4

## Data Availability

The data collected and analyzed in the present study have been presented in the figures of this manuscript or are provided in the figures and tables in the Supplementary Materials.

## Supplementary Materials

The following supporting information can be downloaded at: https://www.mdpi.com/article/10.3390/mps8030056/s1, Methods; Figure S1: Sample to sample variation observed with different urine samples as measured via the kynurenine (L-Kyn) colorimetric assay; Figure S2: Specific metabolites interfere with the kynurenine (L-Kyn) colorimetric assay; Figure S3: Nuclear magnetic resonance (^1^H-NMR) spectrum of commercial pooled urine before and after the treatment with IRA 400 resin, a strong anionic exchanger resin; Figure S4: Differences in L-kynurenine (L-Kyn) levels in spiked and unspiked Canadian urine samples calculated from calibration curves generated with and without background subtraction; Figure S5: Kynurenine (L-Kyn) quantified by liquid chromatography mass spectrometry (LC-MS) in urine samples obtained from Nigerian participants (see Zheng et al., 2023 [30]). Table S1: Metabolites and their concentrations tested for interference in the kynurenine assay; Table S2: L-Kynurenine (L-Kyn) concentrations from urine samples obtained from Nigerian colorectal cancer (CRC) study participants determined by colorimetric assay or by liquid chromatography tandem mass spectrometry (LC-MS/MS).

## Abbreviations

The following abbreviations are used in this manuscript:

AHR	aryl hydrocarbon receptor
CNT	carbon nanotube
C-PU	commercial pooled urine
CRC	colorectal cancer
DAS	diacetylspermine
DFI	direct flow injection
DMAB	*p*-dimethylaminobenzaldehyde
DSS-d_6_	2,2-dimethyl-2-silapentane-5 sulfonate
EtOH	ethanol
ELISA	enzyme-linked immunosorbent assay
3-HK	3-hydroxykynurenine
HPLC	high-performance liquid chromatography
IBS	irritable bowel syndrome
IDO	indoleamine 2,3-dioxygenase
IRA 400	anion exchange resin
kDa	kilodalton
KOAc	potassium acetate
KOH	potassium hydroxide
KP	kynurenine pathway
L-Kyn	kynurenine
LC-MS/MS	liquid chromatography tandem mass spectrometry
LiOH	lithium hydroxide
LLOD	lower limit of detection
LOQ	limit of quantification
L-PU	local pooled urine
NaNO_2_	sodium nitrite
NMR	nuclear magnetic resonance
POC	point-of-care
*p*-TsOH	para-toluenesulfonic acid
TDO	tryptophan 2,3-dioxygenase
Trp	tryptophan
