# Supplementary material for: Towards Automated Testing of Kynurenine for Point-of-Care Metabolomics
Source: Methods Protoc. 2025 Jun 1;8(3):56. doi: 10.3390/mps8030056 (PMC12196141; doi:10.3390/mps8030056)
Supplement: Supplementary file 1 [file mps-08-00056-s001.zip › mps-3616529-supplementary.pdf]

Supplementary Materials

for

Towards Automated Testing of Kynurenine for Point-of-care Metabolomics

Dipanjan Bhattacharyya[a], Marcia LeVatte[a] and David S. Wishart[a,b,c,d*]

[a]Department of Biological Sciences, University of Alberta, Edmonton, AB, T6G 2E9, Canada

[b]Department of Computing Science, University of Alberta, Edmonton, AB, T6G 2E8, Canada

[c]Department of Laboratory Medicine and Pathology, University of Alberta, Edmonton, AB, T6G 1C9, Canada

[d]Faculty of Pharmacy and Pharmaceutical Sciences, University of Alberta, Edmonton, AB, T6G 2H7, Canada

*Corresponding author: Dr. David Wishart

Phone: + 01-780-492-8574

Email: dwishart@ualberta.ca

METHODS

*Preparation of Kynurenine (*L-Kyn) *Calibration Curves in Urine or Serum/Plasma*

For both L-Kyn calibration curves prepared in urine or serum/plasma, a 10 mM L-Kyn stock solution was used. The 10 mM L-Kyn stock solution was prepared by diluting 100 μL of 100 mM L-Kyn (20.82 mg L-Kyn/1 mL 0.5 M HCl) with 900 μL of Milli-Q water. Both the 10 mM and 100 mM L-Kyn stock solutions were stored at −20 °C. For the urine calibration curve, first 200 μL of the 3× diluted PU was aliquoted into six PCR tubes. Then 0, 0.5, 1, 2, 4 and 6 μL of the diluted urine was removed and replaced with the same volume of 10 mM L-Kyn, generating solutions with 0, 25, 50, 100, 200 and 300 μM L-Kyn, respectively.

For the calibration curve prepared in plasma, 100 μL of the pooled deproteinized plasma (SRM 1950) was aliquoted into seven PCR tubes. Then 0, 0.125, 0.25, 0.5, 0.75, 1, and 1.25 μL of plasma was removed and replaced with the same volume of 10 mM L-Kyn, generating solutions with 0, 12.5, 25, 50, 75, 100, and 125 μM L-Kyn, respectively.

Preparation of Solid Potassium Acetate Buffer Cakes

Diazotization reactions typically require acidic pH levels to produce colored reaction products. As the pH of normal urine is between 5.6-6.5, we needed to lower the pH of the urine samples to allow the diazotization reaction to proceed and detect the presence of L-Kyn. This was achieved by adding lyophilized KOAc to the urine prior to passing the sample through the Amberlite® IRA 400 resin. The 0.1 M KOAc (pH 5) was prepared from two solutions: glacial acetic acid (11.55 mL in 1 L Milli-Q water) and KOAc (19.6 g dissolved in 1 L of Milli-Q water). 14.8 mL of the acetic acid solution was mixed with 35.2 mL of KOAc solution in a graduated 100 mL bottle. Then Milli-Q water was added to 100 mL, creating 0.1 M KOAc buffer of pH 5. The pH of the

solution was checked using a pH meter and adjusted by the addition of 0.1 M HCl or 0.1 M KOH as required. Then 250 μL of this buffer solution were aliquoted into 1.5 mL microfuge tubes, frozen at 80 °C for 20 min and lyophilized overnight to create the KOAc buffer cakes.

Removal of Interfering Metabolites from Urine Samples

Urine samples with or without spiked L-Kyn were diluted 3 times (100 μL urine in 200 μL Milli-Q water) and then 200 μL of the diluted urine was aliquoted into one lyophilized KOAc buffer cake. The whole content of the tubes (200 μL) were then transferred to microfuge tubes containing the washed IRA 400 anion exchanger resin. The urine samples were vortexed vigorously and then, for the next 10 min, gently vortexed every 2 min. The resin was centrifuged 19,000 × *g* for 2 min at RT and supernatant (containing L-Kyn) aliquoted into PCR tubes.

Removal of Protein from Serum/Plasma Samples by Ultrafiltration or Precipitation

Before serum or plasma can be assayed for the presence of L-Kyn, abundant proteins, which can interfere with the assay, must be removed by one of two common methods: ultrafiltration or precipitation. To precipitate serum/plasma proteins, 20 μL of 2 M *p*-TsOH solution was added to 200 μL serum in 1.5 mL microfuge tubes, vortexed briefly to mix and incubated for 15 min at RT. The precipitated protein was centrifuged at 19,000 × *g* for 20 min at RT. The supernatant was removed and aliquoted into PCR tubes. To remove serum/plasma proteins by ultrafiltration, 3 kDa Amicon filters were used. To remove the glycerol that is normally added to preserve the Amicon filter membranes, the filters were first washed by centrifuging three times with 500 μL water at 10,000 × *g* for 15 min at 4 °C. Then 500 μL of a serum/plasma sample was loaded onto the washed

filters and centrifuged at 10,000 × *g* for 25 min at 4 °C. The ultrafiltered serum was then aliquoted into PCR tubes.

Kynurenine Assay for Urine or Serum/Plasma

100 μL of the resin-treated urine samples or 100 μL of deproteinized serum/plasma was aliquoted into PCR tubes and stored on ice. Using a pipettor, 10 μL of 0.5 M *p*-TsOH (951 mg *p*-TsOH•H_2O in 10 mL Milli-Q water, stored at –20 °C) was added to the treated urine or serum/plasma samples. This was followed by 10 μL of freshly prepared $NaNO_2$ (70 mg $NaNO_2$ in 1 mL Milli-Q water). The reactants were mixed by repeatedly pipetting the samples up and down four or five times. Afterwards, the mixture was incubated for 2 min at RT under quiescent conditions. Then 30 μL freshly prepared solution of 2-naphthol (14 mg β-naphthol in 1.5 mL 0.5 M LiOH or 0.5 M NaOH) was then added to the reaction mixtures and mixed thoroughly by pipetting up and down. 100 μL of each of the reaction mixtures were then transferred to a 96-well plate. Three min after the addition of 2-naphthol solution, the absorbance at 490 nm (A_{490}) was measured using a BioTek® Synergy HT UV-Vis spectrophotometer (Winooski, USA).

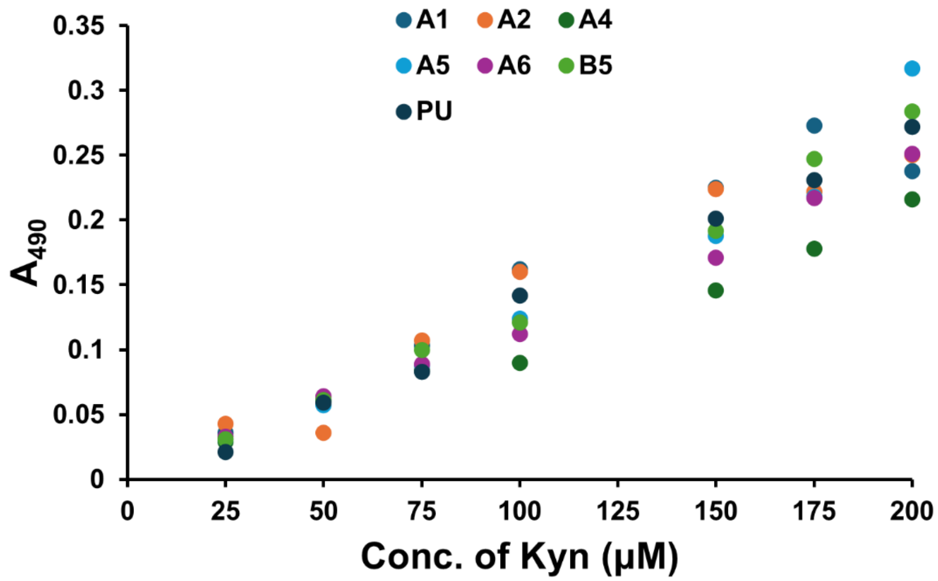

Figure S1. Sample to sample variation observed with different urine samples as measured via the kynurenine (L-Kyn) colorimetric assay. Seven different urine samples from healthy individuals, diluted 3×, spiked with increasing concentrations of L-Kyn, were processed via the colorimetric L-Kyn assay and absorbance readings at 490 nm were plotted against the spiked-in concentrations of L-Kyn. High variability of reaction was seen with the different urine samples suggesting that urinary metabolites may be interfering with the chemical assay.

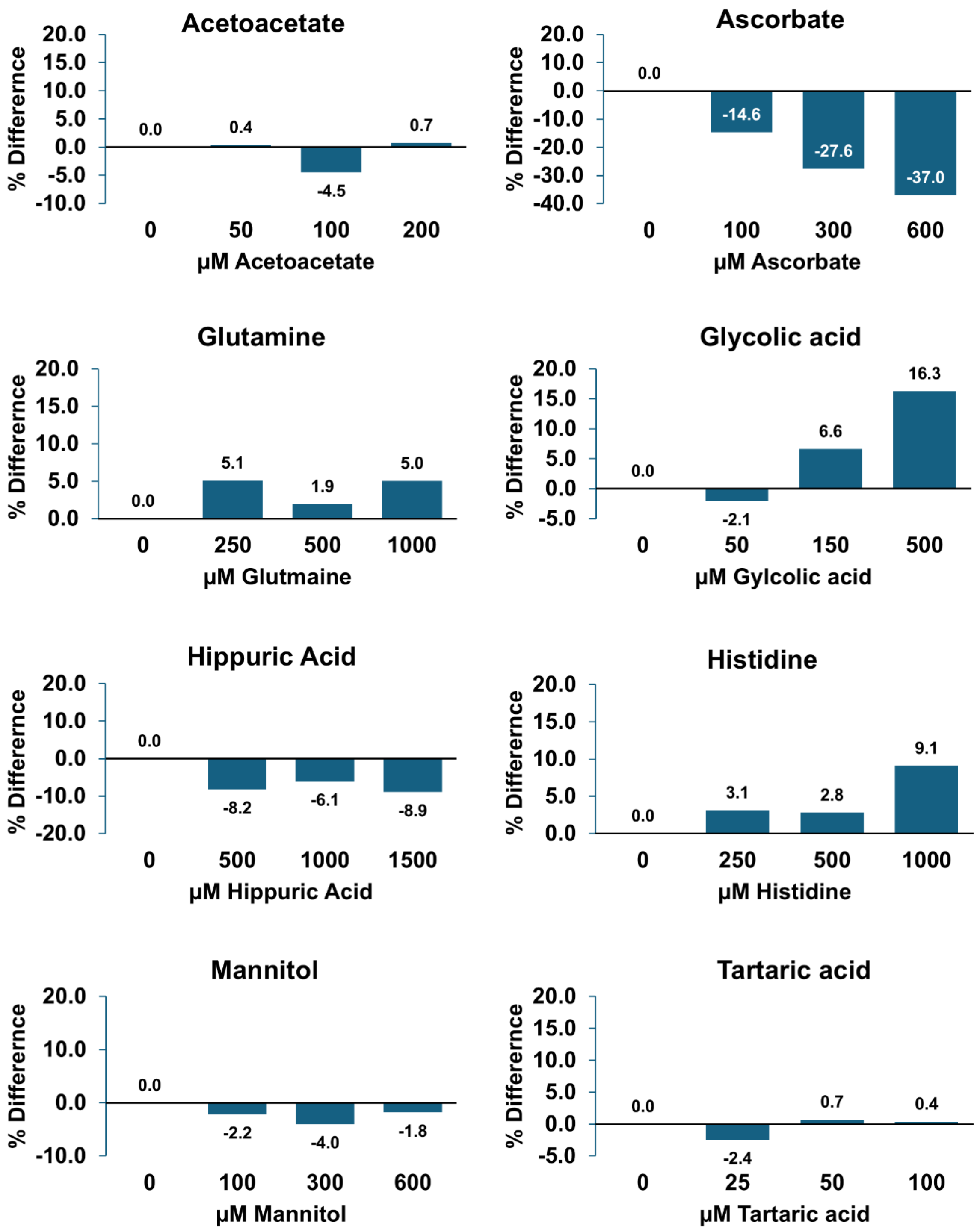

Figure S2. Specific metabolites interfere with the kynurenine (L-Kyn) colorimetric assay. Increasing concentrations of the abundant urinary metabolites (levels added were based on the expected normal urinary values reported in the Human Metabolome Database (HMDB) were

prepared in the solutions indicated in Table S1, added to 100 µM Kyn spiked into water and assayed by the L-Kyn assay. The difference in absorbance obtained for 100 µM L-Kyn was subtracted from the absorbance of 100 µM L-Kyn spiked with different concentrations of each metabolite, divided by the absorbance of 100 µM L-Kyn, then multiplied by 100 (see equation 1 in the main text). Ascorbic acid interference was the greatest, reducing the absorbance by up to 37% when 600 µM was added. Other metabolites that decreased absorbance were acetoacetate, hippuric acid, mannitol and tartaric acid. Additions of glutamine, histidine and glycolic acid led to increased absorbances.

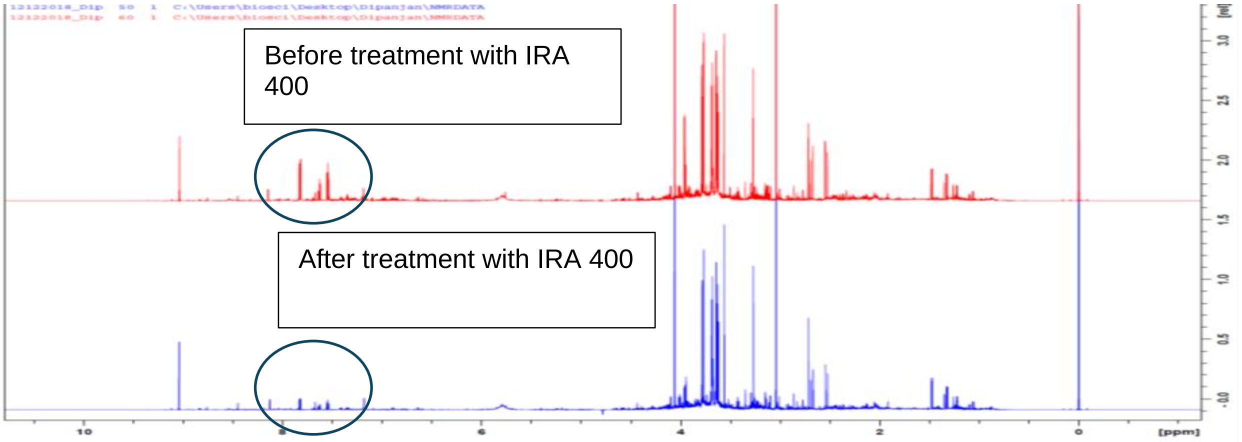

Figure S3. Nuclear magnetic resonance (^{1}H-NMR) spectrum of commercial pooled urine before and after the treatment with IRA 400 resin, a strong anionic exchanger resin. The section of spectra that is circled shows that hippuric acid is removed after treatment with resin.

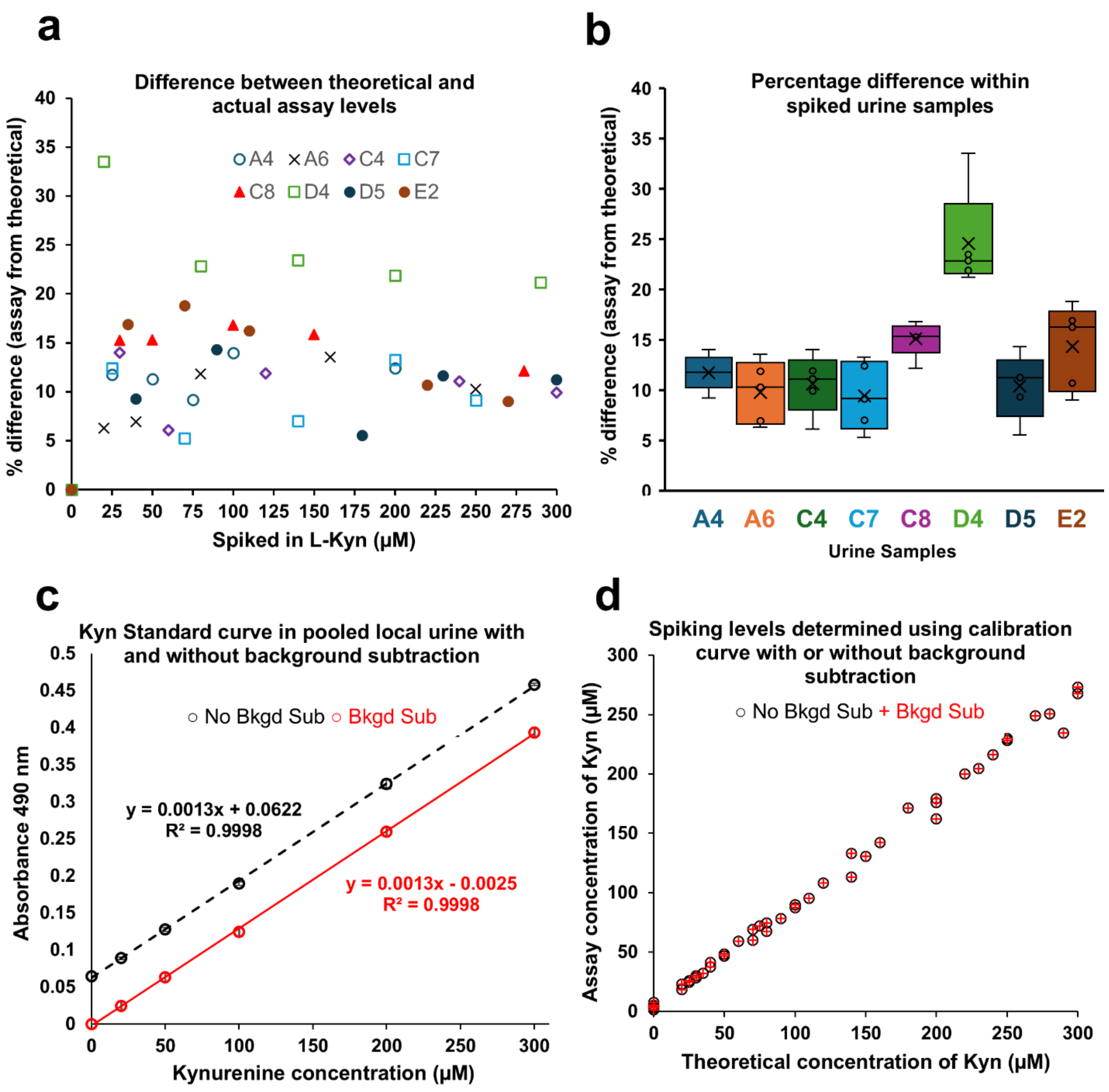

Figure S4. Differences in L-kynurenine (L-Kyn) levels in spiked and unspiked Canadian urine samples calculated from calibration curves generated with and without background subtraction. a) Eight different Canadian volunteer urine samples were spiked with 0-300 µM L-Kyn and assayed with levels determined from the equation of the linear regression of the calibration curve shown in panel c (without background subtraction). As unspiked samples were also assayed, the theoretical levels were the sum of the endogenous L-Kyn plus the spiked-in

concentration. The percentage difference was calculated by subtracting the theoretical concentration from the assayed level, dividing by the theoretical concentration and then multiplying by 100 (see equation below). The percentage difference ranged from 5.3-33.7 %, averaging 13.9 ± 5.66 % difference. This difference did not increase with the higher spiked-in levels of L-Kyn. b) Box and whisker plots of the percentage differences in the eight urine samples shows that most samples average 10-15 % differences with only one sample (D4) having 23% average difference. c) Calibration curve generated when the absorbance of the spiked or unspiked commercial pooled urine (C-PU) standards with and without background subtraction (of the unspiked sample) were plotted against L-Kyn spiked into the 3× diluted C-PU. Note that the coefficients of determination (R^2) are identical but that the equations of linear regression differ only by amount of absorbance added (+ 0.0622) or subtracted (-0.0025). d) When the absorbance of Canadian volunteer urine samples were spiked with 0 - 300 μM L-Kyn and their levels determined from the equations of the linear regression (see below), the results were the same. Background subtraction did not impact the data. Abbreviations: Bkgd – background; Sub – subtraction

$$\% \, difference = \frac{(\text{μM theoretical} - \text{μM assay})}{\text{μM theoretical}} \times 100$$

No background subtraction: $$x\ (\text{μM}) = \frac{y\ (unknown\ absorbance) - 0.0622}{0.0013}$$

Background subtraction: $$x\ (\text{μM}) = \frac{y\ (unknown\ absorbance) + 0.0025}{0.0013}$$

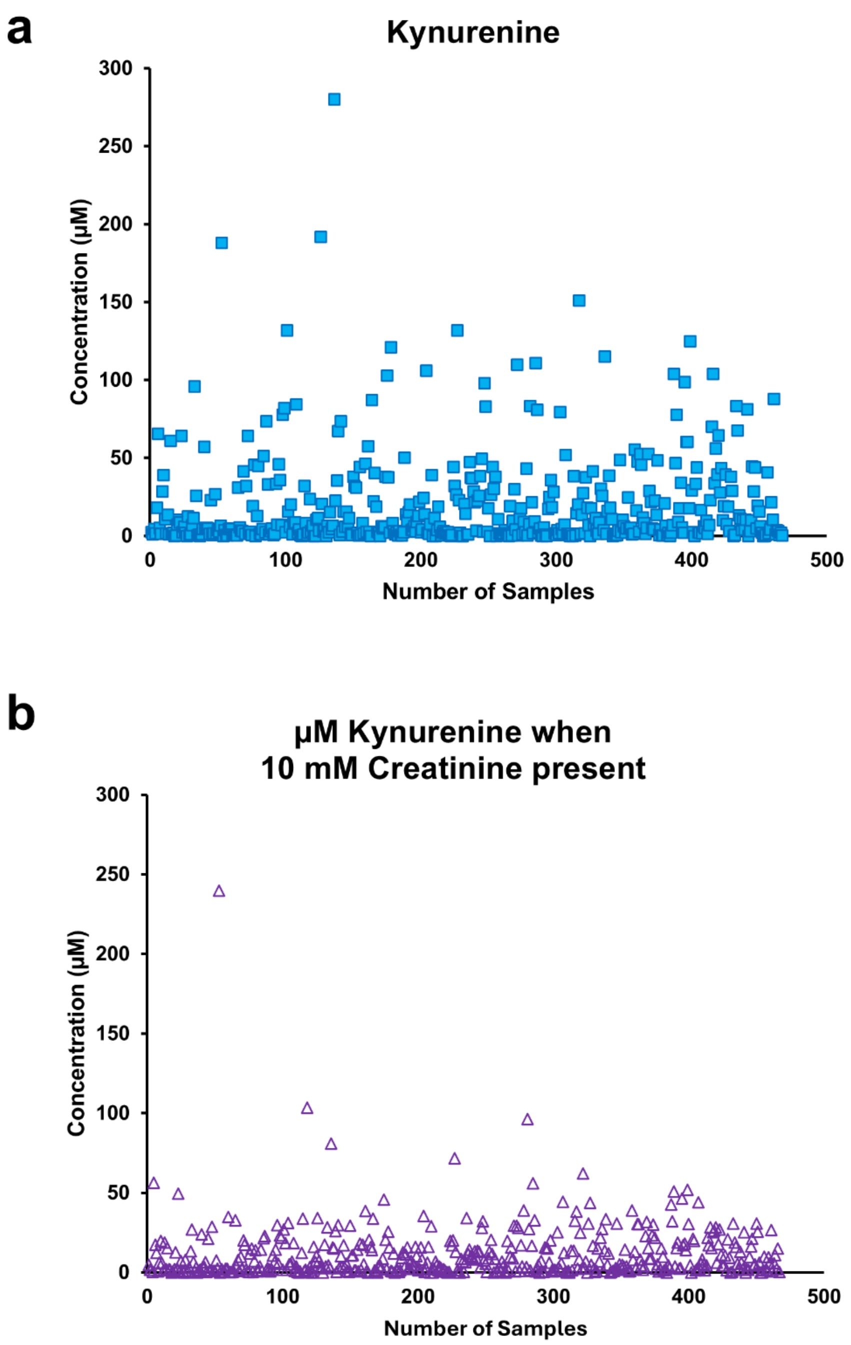

Figure S5. Kynurenine (L-Kyn) quantified by liquid chromatography mass spectrometry (LC-MS) in urine samples obtained from Nigerian participants (see Zheng et al, 2023). a) Concentration of L-Kyn in 467 urine samples not normalized for creatinine concentrations. b) Concentration of L-Kyn in urine samples normalized for creatinine concentrations, calculated by dividing the L-Kyn levels (µM) by the creatinine concentration (mM) and then multiplying by 10.

Table S1. Metabolites and their concentrations tested for interference in the kynurenine assay

Number	Metabolite (Solvent)[a]	Concentration ranges in urine (avg.) µM/mM of creatinine[b]	Tested Concentrations (µM)
1	2-Aminoisobutyric Acid (water)	2.2-140 (26) 3-Aminoisobutyric Acid	100, 250, 500
2	Allantoin (0.5 M LiOH)	4.9-29.3 (15.4)	50, 150, 300
3	Arabinitol (water)	10.2-64.3 (31.8)	100, 300, 600
4	**Ascorbic Acid (water)** [c]	**4.6-78 (32.5)**	**100, 300, 600**
5	Citric Acid (water)	49-600 (203)	250, 500, 1000
6	Creatine (0.5 M LiOH)	3-448 (46)	250, 500, 1000
7	Glucose (water)	12.5-58.4 (37.5)	100, 250, 500
8	Ethanolamine HCl salt (water)	24.8-56.2 (37)	100, 250, 500
9	Glycine (water)	44-300 (106)	500, 1000, 1500
10	**Glycolic Acid (water)**	**3.7-122 (42)**	**50, 150, 500**
11	**Hippuric Acid (1 M LiOH)**	**19-622 (229)**	**500, 1000, 1500**
12	Cysteine (water)	23.1-134.5 (65.8)	250, 500, 1000
13	**Glutamine (0.6 M HCl)**	**19.1-77.9 (37.2)**	**250, 500, 1000**
14	**Histidine (0.6 M HCl)**	**17-90 (43)**	**250, 500, 1000**
15	Tryptophan (0.5 M LiOH)	3.4-11.1 (6.3)	25, 50, 100
16	Tyrosine (0.6 M HCl)	4.1-23.5 (9.5)	50, 100, 200
17	**Mannitol (water)**	**5.2-85.1 (32.4)**	**100, 300, 600**

18	**Tartaric Acid (0.5 M LiOH)**	**2.6-64.4 (11.8)**	**25, 50, 100**
19	Taurine (0.5 M LiOH)	13-251 (81)	250, 500, 1000
20	Urea (water)	174-49097 (12285)	100 mM, 200 mM, 400 mM
21	**Lithium Acetoacetate (water)**	**2.2-24.9 (11.1)**	**50, 100, 200**

[a] Metabolites and their indicated concentrations, prepared in the indicated solutions, were added to 100 μM kynurenine prepared in water and assayed using the optimized kynurenine assay.

[b] Values found in the Human Metabolome Database or HMDB (https://hmdb.ca/).

[c] Metabolites that are **bolded** interfered with the assay. See **Figure S2** for graphed results of interfering metabolites and their differences.

Table S2. L-Kynurenine (L-Kyn) concentrations from urine samples obtained from Nigerian colorectal cancer (CRC) study participants determined by colorimetric assay or by liquid-chromatography tandem mass spectrometry (LC-MS/MS)

Sample number	L-Kyn Assay (μM)	LC-MS/MS (μM)
1.	137.1	83.2
2.	85.4	37.6
3.	160.5	77.9
4.	56.4	40.5
5.	310.0	280
6.	91.6	48.3
7.	97.3	38.6
8.	69.9	52.7
9.	159.2	111
10.	96.0	83.4
11.	134.9	84.4
12.	159.6	121
13.	196.3	196
